# Alzheimer’s Disease and Lewy Body Co‐Pathology Presenting with Corticobasal Syndrome

**DOI:** 10.1002/alz.088634

**Published:** 2025-01-03

**Authors:** Claire Delpirou Nouh, Kristen Vallejo, Simon Tan, Michael Zeineh, Sharon J Sha, Jeffrey Nirschl

**Affiliations:** ^1^ Department of Neurology and Neurological Sciences, Stanford University, Stanford, CA USA; ^2^ Stanford University School of Medicine, Stanford, CA USA; ^3^ Stanford University, Stanford, CA USA

## Abstract

**Background:**

Corticobasal syndrome (CBS) corresponds to a clinical phenotype with heterogeneous neuropathology, including corticobasal degeneration (CBD), progressive supranuclear palsy (PSP), Alzheimer’s disease (AD), and synucleinopathies such as Lewy Body Disease (LBD), in rare cases. Previous reports of CBS‐LBD describe patients with diffuse LBD, a younger age of onset and occasionally lacking core features like REM sleep Behavior Disorder (RBD).

**Method:**

We present a young patient with CBS who had a rapid progression and was found to have a high burden of limbic LBD and high AD co‐pathology at autopsy.

**Result:**

A 58‐year‐old right‐handed woman consulted for progressively worsening apraxia, writing, and visuospatial difficulties over the year prior to presentation. She had minimal short‐term memory loss initially, did not have RBD nor visual hallucinations and remained independent in activities of daily living. She scored 20/30 on the Montreal Cognitive Assessment, with difficulties in visuospatial and executive tasks. The neurological exam showed predominant apraxia, asymmetric parkinsonism and sensory neglect, all left‐sided. The brain MRI confirmed striking asymmetric atrophy affecting the right parieto‐temporal lobes. Cerebrospinal fluid (CSF) demonstrated decreased Abeta 42/tau index and elevated total and phosphorylated tau. Her condition progressed quickly over the next year, and she passed away four years after diagnosis. Brain autopsy showed asymmetric moderate‐severe cortical atrophy with abundant phospho‐synuclein positive Lewy bodies extending to the temporal but not frontal/parietal cortices, consistent with limbic (transitional) LBD and high Alzheimer’s disease co‐pathology (A3, B3, C3). There was severe Lewy body pathology in the hippocampal CA2 subfield, prominent phospho‐tau in the substantia nigra, severe neuron loss in the locus coeruleus, and severe gliosis in the motor cortex.

**Conclusion:**

AD and LBD commonly occur together, but the frequency of AD and LBD as a cause of CBS is unknown. Here, we add to the literature of co‐pathology in CBS and consider that early age and rapid progression could suggest dual pathology and potential synergistic effects. CSF testing limited to AD biomarkers will miss this dual pathology. Thus, CSF α‐synuclein seeding amplification may help identify patients with dual AD‐LBD pathology and assist prognostication.

